# Supplementary material for: Depression with atypical neurovegetative symptoms shares genetic predisposition with immuno-metabolic traits and alcohol consumption
Source: Psychol Med. 2020 Jul 6;52(4):726–36. doi: 10.1017/S0033291720002342 (PMC8961332; doi:10.1017/S0033291720002342)
Supplement: Supplementary file 1 [file S0033291720002342sup001.pdf]

## Supplementary Materials

# Depression with atypical neurovegetative symptoms shares genetic predisposition with immuno-metabolic traits and alcohol consumption

**Supplementary Table 1**: genome-wide association studies (GWAS) used to create polygenic risk scores (PRS) ........................................................................................ p. 2

**Supplementary Table 2**: PRS results for ↑WS depression vs. depression without ↑WS...... p. 3

**Supplementary Table 3**: PRS results for ↑WS depression vs. ↓WS depression .................. p. 5

**Supplementary Table 4**: PRS results for ↑WS depression vs. healthy controls ................... p. 7

**Supplementary Table 5**: PRS results for ↓WS depression vs. healthy controls ................... p. 9

**Supplementary Figure 1**: PRS results at the different $P_T$ ...................................................... p. 11

**Supplementary Figure 2**: PRS results for ↑WS depression vs. non-↑WS depression after excluding people with probable bipolar disorder or schizophrenia............................................ p. 14

**Supplementary Figure 3**: PRS results for ↑WS depression vs. ↓WS depression after excluding people with probable bipolar disorder or schizophrenia............................................ p. 15

**Supplementary Figure 4**: PRS results for ↑WS depression and ↓WS depression vs. healthy controls after excluding people with probable bipolar disorder or schizophrenia......... p. 16

**Supplementary Figure 5**: GCTB-BayesS estimates of S and Pi parameters............................ p. 17

**References**........................................................................................................................ p. 18

**Supplementary Table 1:** Summary of genome-wide association studies (GWAS) used to create polygenic risk scores.

| Trait | Type of phenotype | N cases | N controls | Ethnicity | Reference |
|---|---|---|---|---|---|
| **Psychiatric disorders** | | | | | |
| Major depressive disorder** | Binary | 45,591 | 97,674 | European | (Wray et al., 2018) |
| Bipolar disorder | Binary | 20,352 | 31,358 | European | (Stahl et al., 2019) |
| Schizophrenia | Binary | 36,989 | 113,075 | European | (Ripke et al., 2014) |
| Anxiety disorders | Binary | 7,016 | 14,745 | European | (Otowa et al., 2016) |
| Alcohol dependence | Binary | 14,904 | 37,944 | Trans-ethnic | (Walters et al., 2018) |
| PTSD | Binary | 5,131 | 15,092 | Trans-ethnic | (Duncan et al., 2018) |
| Anorexia nervosa* | Binary | 16,992 | 55,525 | European | (Watson et al., 2019) |
| **Alcohol use and smoking** | | | | | |
| Alcohol daily use | Continuous | 70,460 | | European | (Schumann et al., 2016) |
| N cigarettes per day | Continuous | 38,181 | | European | (Tobacco and Genetics Consortium, 2010) |
| Cannabis use lifetime | Binary | 14,374 | 17,956 | European | (Stringer et al., 2016) |
| **Personality traits** | | | | | |
| Extraversion | Continuous | 63,030 | | European | (van den Berg et al., 2016) |
| Neuroticism | Continuous | 63,030 | | European | (Genetics of Personality Consortium et al., 2015) |
| **Cardio-metabolic traits** | | | | | |
| Type 2 diabetes | Binary | 26,676 | 132,532 | Trans-ethnic | (Scott et al., 2017) |
| Coronary artery disease | Binary | 60,801 | 123,504 | European and south Asian | (Nikpay et al., 2015) |
| Triglycerides | Continuous | 188,577 | | Trans-ethnic | (Willer et al., 2013) |
| Total LDL cholesterol | Continuous | 188,577 | | Trans-ethnic | (Willer et al., 2013) |
| Total HDL cholesterol | Continuous | 188,577 | | Trans-ethnic | (Willer et al., 2013) |
| BMI | Continuous | 322,154 | | European | (Locke et al., 2015) |
| Leptin | Continuous | 31,816 | | European | (Kilpeläinen et al., 2016) |
| Leptin adjusted for BMI | Continuous | 31,816 | | European | (Kilpeläinen et al., 2016) |
| CRP | Continuous | 204,402 | | European | (Ligthart et al., 2018) |
| Ischemic stroke | Binary | 67,162 | 454,450 | Trans-ethnic | (Malik et al., 2018) |

** Subjects included in UK Biobank and 23AndMe were excluded from the analyses
* This sample had a marginal overlap (2% of cases) with UK biobank depressed sample included in these analyses

**Supplementary Table 2: A.** PRS results for ↑WS depression (n=1,854) vs. depression without ↑WS (n=28,215) (including bipolar disorder and/or schizophrenia cases, the results were similar when excluding these groups, see Supplementary Figure 2). **B.** Nagelkerke R2 was reported on the observed scale (prevalence of ↑WS depression among people with major depression in UK Biobank = 0.066) and considering other plausible prevalence values of ↑WS depression among people with major depression (these comparisons included only cases and not healthy controls). **C.** Results obtained by comparing individual neurovegetative symptoms for the significant PRS and leptin adjusted for BMI for comparison with the main results. Significant results are in bold.

**A.**

| Trait | Best p threshold | Beta (SE) | P | OR (95% CI) |
|---|---|---|---|---|
| **Major depressive disorder** | 0.3 | 0.09 (0.02) | 1.14e-04** | 1.10 (1.05-1.15) |
| Bipolar disorder | 1e.5 | 0.05 (0.02) | 0.05 | 1.05 (1-1.10) |
| Schizophrenia | 0.01 | 0.03 (0.02) | 0.15 | 1.04 (0.99-1.09) |
| Anxiety disorders | 0.3 | 0.04 (0.02) | 0.13 | 1.04 (0.99-1.09) |
| Alcohol dependence | 1e-05 | -0.05 (0.02) | 0.04* | 0.95 (0.91-0.99) |
| PTSD | 0.01 | 0.05 (0.02) | 0.03* | 1.05 (1-1.10) |
| Anorexia nervosa | 0.4 | -0.04 (0.02) | 0.08 | 0.96 (0.91-1) |
| **Alcohol daily use** | 0.4 | -0.13 (0.03) | 1.04e-05** | 0.88 (0.83-0.93) |
| N cigarettes per day | 5e-08 | 0.04 (0.02) | 0.08 | 1.04 (0.99-1.09) |
| Cannabis use lifetime | 1e-05 | 0.03 (0.02) | 0.14 | 1.04 (0.99-1.09) |
| Extraversion | 0.001 | 0.03 (0.02) | 0.28 | 1.03 (0.98-1-08) |
| Neuroticism | 0.001 | -0.03 (0.02) | 0.27 | 0.97 (0.93-1.02) |
| Type 2 diabetes | 0.001 | 0.07 (0.02) | 0.005* | 1.07 (1.02- 1.12) |
| Coronary artery disease | 0.5 | 0.07 (0.02) | 0.007* | 1.07 (1.02- 1.12) |
| Triglycerides | 1 | 0.06 (0.02) | 0.02* | 1.06 (1.01-1.11) |
| Total LDL cholesterol | 1e-05 | -0.04 (0.02) | 0.08 | 0.96 (0.92-1) |
| Total HDL cholesterol | 0.3 | -0.06 (0.02) | 0.02* | 0.94 (0.90-0.99) |
| **BMI** | 0.001 | 0.18 (0.02) | 2.37e-14** | 1.20 (1.15-1.26) |
| **Leptin** | 0.001 | 0.09 (0.02) | 2.99e-04* | 1.09 (1.04-1.14) |
| Leptin adjusted for BMI | 0.3 | 0.03 (0.02) | 0.28 | 1.03 (0.98-1.08) |
| **CRP** | 0.4 | 0.11 (0.02) | 8.86e-06** | 1.11 (1.06-1.17) |
| Ischemic stroke | 0.4 | 0.06 (0.02) | 0.01* | 1.06 (1.01-1.12) |

* $p < 0.05$; ** $p < 2.1e-04$

**B.**

| Trait | Observed scale (K=0.066) | K=0.10 | K=0.15 | K=0.20 |
|---|---|---|---|---|
| **Major depressive disorder** | 0.00133 | 0.00224 | 0.00255 | 0.00278 |
| Bipolar disorder | 0.00035 | 0.00059 | 0.00067 | 0.00074 |
| Schizophrenia | 0.00018 | 0.00031 | 0.00035 | 0.00038 |
| Anxiety disorders | 0.00020 | 0.00034 | 0.00038 | 0.00042 |
| Alcohol dependence | 0.00037 | 0.00062 | 0.00070 | 0.00078 |
| PTSD | 0.00041 | 0.00069 | 0.00079 | 0.00086 |
| Anorexia nervosa | 0.00028 | 0.00047 | 0.00053 | 0.00058 |
| **Alcohol daily use** | 0.00174 | 0.00292 | 0.00332 | 0.00363 |

| | | | | |
|---|---|---|---|---|
| N cigarettes per day | 0.00027 | 0.00046 | 0.00052 | 0.00057 |
| Cannabis use lifetime | 0.00020 | 0.00033 | 0.00038 | 0.00041 |
| Extraversion | 0.00010 | 0.00018 | 0.00020 | 0.00022 |
| Neuroticism | 0.00011 | 0.00018 | 0.00020 | 0.00022 |
| Type 2 diabetes | 0.00071 | 0.00119 | 0.00135 | 0.00148 |
| Coronary artery disease | 0.00064 | 0.00108 | 0.00123 | 0.00134 |
| Triglycerides | 0.00051 | 0.00086 | 0.00097 | 0.00106 |
| Total LDL cholesterol | 0.00027 | 0.00046 | 0.00052 | 0.00057 |
| Total HDL cholesterol | 0.00052 | 0.00088 | 0.0010 | 0.00109 |
| **BMI** | 0.00520 | 0.00875 | 0.00994 | 0.01085 |
| **Leptin** | 0.00117 | 0.00197 | 0.00224 | 0.00244 |
| Leptin adjusted for BMI | 0.00010 | 0.00017 | 0.00020 | 0.00022 |
| **CRP** | 0.00177 | 0.00297 | 0.00338 | 0.00369 |
| Ischemic stroke | 0.00055 | 0.00093 | 0.00106 | 0.00116 |

**C.**

**Depression with weight increase (n=5,801) vs. depression without weight increase (n=20,561):**

| **Trait** | **Best p threshold** | **Beta (SE)** | **P** | **OR (95% CI)** |
|---|---|---|---|---|
| Major depressive disorder | 0.3 | 0.04 (0.02) | 4.97e-03* | 1.04 (1.01-1.07) |
| Alcohol daily use | 1 | -0.07 (0.02) | 1.99e-04** | 0.93 (0.90-0.97) |
| BMI | 0.4 | 0.24 (0.02) | 2.53e-56** | 1.28 (1.24-1.32) |
| Leptin | 0.1 | 0.08 (0.01) | 6.43e-08** | 1.08 (1.05-1.12) |
| Leptin adjusted for BMI | 0.4 | 0.01 (0.01) | 0.61 | 1.01 (0.98-1.04) |
| CRP | 0.4 | 0.11 (0.02) | 3.09e-13** | 1.12 (1.08-1.15) |

**Depression with hypersomnia (n=6,923) vs. depression without hypersomnia (n=18,871):**

| **Trait** | **Best p threshold** | **Beta (SE)** | **P** | **OR (95% CI)** |
|---|---|---|---|---|
| Major depressive disorder | 0.3 | 0.05 (0.01) | 3.98e-04* | 1.05 (1.02-1.08) |
| Alcohol daily use | 0.05 | -0.03 (0.02) | 0.10 | 0.97 (0.95-1.01) |
| BMI | 1 | 0.03 (0.01) | 0.046* | 1.03 (1.00-1.06) |
| Leptin | 0.2 | 0.03 (0.01) | 0.070 | 1.03 (1.00-1.05) |
| Leptin adjusted for BMI | 1e-05 | -0.01 (0.01) | 0.48 | 0.99 (0.96-1.02) |
| CRP | 0.1 | 0.05 (0.01) | 9.94e-04* | 1.05 (1.02-1.08) |

**Supplementary Table 3: A.** PRS results for ↑WS depression (n=1,854) vs. ↓WS depression (n=10,142) (including bipolar disorder and/or schizophrenia cases, the results were similar when excluding these groups, see Supplementary Figure 3). **B.** Nagelkerke R2 is reported on the observed scale (prevalence K=0.15 in UK Biobank) and according to different plausible values of prevalence of ↑WS depression among people having depression with neurovegetative symptoms (these comparisons included only cases and not healthy controls). Significant results are in bold.

**A**

| Trait | Best p threshold | Beta (SE) | P | OR (95% CI) |
|---|---|---|---|---|
| Major depressive disorder | 0.3 | 0.08 (0.03) | 1.10e-03* | 1.09 (1.03-1.14) |
| Bipolar disorder | 0.4 | 0.04 (0.03) | 0.12 | 1.04 (0.99-1.10) |
| Schizophrenia | 5e.8 | 0.02 (0.03) | 0.38 | 1.02 (0.97-1.08) |
| Anxiety disorders | 0.3 | 0.02 (0.03) | 0.39 | 1.02 (0.97-1.07) |
| Alcohol dependence | 1e-05 | -0.06 (0.03) | 0.03* | 0.95 (0.89-0.99) |
| PTSD | 0.01 | 0.05 (0.03) | 0.05 | 1.05 (1.00-1.11) |
| Anorexia nervosa | 0.4 | -0.06 (0.03) | 0.02* | 0.94 (0.89-0.99) |
| **Alcohol daily use** | 0.4 | -0.14 (0.03) | 1.34e-05** | 0.87 (0.82-0.93) |
| N cigarettes per day | 5e-8 | 0.04 (0.03) | 0.11 | 1.04 (0.99-1.10) |
| Cannabis use lifetime | 1e-5 | 0.03 (0.03) | 0.20 | 1.03 (0.98-1.08) |
| Extraversion | 0.001 | 0.03 (0.03) | 0.18 | 1.03 (0.98-1.09) |
| Neuroticism | 0.001 | 0.03 (0.03) | 0.18 | 0.97 (0.92-1.02) |
| Type 2 diabetes | 0.001 | 0.08 (0.03) | 0.0022* | 1.08 (1.03-1.14) |
| Coronary artery disease | 0.5 | 0.07 (0.03) | 0.012* | 1.07 (1.01-1.12) |
| Triglycerides | 1 | 0.06 (0.03) | 0.019* | 1.06 (1.01-1.12) |
| Total LDL cholesterol | 1e-05 | -0.06 (0.03) | 0.022* | 0.94 (0.90-0.99) |
| Total HDL cholesterol | 0.3 | -0.06 (0.03) | 0.023* | 0.94 (0.90-0.99) |
| **BMI** | 0.001 | 0.24 (0.03) | 1.80e-20** | 1.27 (1.21-1.33) |
| Leptin | 0.3 | 0.09 (0.03) | 6.01e-04* | 1.09 (1.04-1.15) |
| Leptin adjusted for BMI | 0.4 | 0.02 (0.03) | 0.35 | 1.02 (0.97-1.08) |
| **CRP** | 0.1 | 0.12 (0.03) | 7.33e-06** | 1.12 (1.07-1.18) |
| Ischemic stroke | 0.4 | 0.06 (0.03) | 0.016* | 1.07 (1.01-1.12) |

* p < 0.05; ** p < 2.1e-04

**B**

| Trait | Observed scale (K=0.15) | K=0.20 | K=0.30 | K=0.40 |
|---|---|---|---|---|
| Major depressive disorder | 0.00152 | 0.00219 | 0.00245 | 0.00259 |
| Bipolar disorder | 0.00034 | 0.00049 | 0.00055 | 0.00058 |
| Schizophrenia | 0.00011 | 0.00016 | 0.00018 | 0.00019 |
| Anxiety disorders | 0.00010 | 0.00015 | 0.00017 | 0.00018 |
| Alcohol dependence | 0.00067 | 0.00097 | 0.00109 | 0.00115 |
| PTSD | 0.00055 | 0.00080 | 0.00089 | 0.00094 |
| Anorexia nervosa | 0.00077 | 0.00111 | 0.00124 | 0.00131 |

| | | | | |
|---|---|---|---|---|
| **Alcohol daily use** | 0.00271 | 0.00391 | 0.00436 | 0.00461 |
| N cigarettes per day | 0.00037 | 0.00053 | 0.00060 | 0.00063 |
| Cannabis use lifetime | 0.00023 | 0.00033 | 0.00037 | 0.00039 |
| Extraversion | 0.00025 | 0.00037 | 0.00041 | 0.00043 |
| Neuroticism | 0.00026 | 0.00037 | 0.00042 | 0.00044 |
| Type 2 diabetes | 0.00134 | 0.00193 | 0.00215 | 0.00228 |
| Coronary artery disease | 0.00089 | 0.00128 | 0.00143 | 0.00152 |
| Triglycerides | 0.00078 | 0.00113 | 0.00126 | 0.00133 |
| Total LDL cholesterol | 0.00075 | 0.00108 | 0.00121 | 0.00127 |
| Total HDL cholesterol | 0.00074 | 0.00106 | 0.00119 | 0.00125 |
| **BMI** | 0.01237 | 0.01783 | 0.01987 | 0.02098 |
| **Leptin** | 0.00168 | 0.00243 | 0.00271 | 0.00286 |
| Leptin adjusted for BMI | 0.00013 | 0.00018 | 0.00020 | 0.00021 |
| **CRP** | 0.00287 | 0.00414 | 0.00463 | 0.00489 |
| Ischemic stroke | 0.00083 | 0.00119 | 0.00133 | 0.00141 |

**Supplementary Table 4: A.** PRS results for ↑WS depression (n=1,854) vs. healthy controls (n=8,000) (including bipolar disorder and/or schizophrenia cases, the results were similar when excluding these groups, see Supplementary Figure 4). **B.** Nagelkerke R2 is reported on the liability scale according to different possible values of ↑WS depression prevalence in the population. Significant results are in bold.

**A**

| Trait | Best p threshold | Beta (SE) | P | OR (95% CI) |
|---|---|---|---|---|
| **Major depressive disorder** | 0.3 | 0.27 (0.02) | 2.39e-28** | 1.30 (1.24-1.36) |
| **Bipolar disorder** | 0.4 | 0.14 (0.02) | 3.04e-08** | 1.15 (1.09-1.20) |
| **Schizophrenia** | 0.4 | 0.15 (0.02) | 4.93e-09** | 1.15 (1.10-1.21) |
| Anxiety disorders | 0.3 | 0.08 (0.02) | 3.70e-04* | 1.09 (1.04-1.14) |
| Alcohol dependence | 0.5 | 0.06 (0.02) | 0.02* | 1.06 (1.01-1.11) |
| PTSD | 0.01 | 0.07 (0.02) | 5.63e-03* | 1.07 (1.02-1.12) |
| Anorexia nervosa | 0.2 | 0.06 (0.02) | 0.01* | 1.06 (1.01-1.11) |
| **Alcohol daily use** | 0.2 | -0.12 (0.03) | 9.31e-06** | 0.88 (0.84-0.93) |
| N cigarettes per day | 0.05 | 0.05 (0.02) | 0.05 | 1.05 (1.00-1.10) |
| Cannabis use lifetime | 0.3 | 0.04 (0.03) | 0.11 | 1.04 (0.99-1.09) |
| Extraversion | 0.2 | -0.03 (0.02) | 0.24 | 0.97 (0.93-1.02) |
| Neuroticism | 0.4 | 0.07 (0.02) | 4.87e-03* | 1.07 (1.02-1.12) |
| Type 2 diabetes | 0.001 | 0.07 (0.02) | 4.70e-03* | 1.07 (1.02-1.12) |
| **Coronary artery disease** | 0.5 | 0.10 (0.02) | 1.60e-05** | 1.11 (1.06-1.16) |
| **Triglycerides** | 1 | 0.09 (0.02) | 8.27e-05** | 1.10 (1.05-1.15) |
| Total LDL cholesterol | 5e-08 | -0.04 (0.02) | 0.12 | 0.96 (0.92-1.01) |
| Total HDL cholesterol | 0.3 | -0.07 (0.02) | 3.84e-03* | 0.93 (0.89-0.98) |
| **BMI** | 0.3 | 0.21 (0.02) | 1.65e-18** | 1.24 (1.18-1.30) |
| **Leptin** | 0.05 | 0.10 (0.02) | 3.01e-05** | 1.10 (1.05-1.16) |
| Leptin adjusted for BMI | 0.5 | 0.03 (0.02) | 0.23 | 1.03 (0.98-1.08) |
| **CRP** | 0.5 | 0.13 (0.02) | 1.21e-07** | 1.13 (1.08-1.19) |
| Ischemic stroke | 0.4 | 0.08 (0.02) | 1.68e-03* | 1.08 (1.03-1.13) |

* $p < 0.05$; ** $p < 2.1e-04$

**B**

| Trait | K=0.01 | K=0.02 | K=0.03 | K=0.04 | K=0.05 |
|---|---|---|---|---|---|
| **Major depressive disorder** | 0.00936 | 0.0111 | 0.01240 | 0.01345 | 0.01436 |
| **Bipolar disorder** | 0.00235 | 0.00279 | 0.00311 | 0.00338 | 0.00361 |
| **Schizophrenia** | 0.00262 | 0.00312 | 0.00348 | 0.00377 | 0.00403 |
| Anxiety disorders | 0.00097 | 0.00115 | 0.00129 | 0.00140 | 0.00149 |
| Alcohol dependence | 0.00040 | 0.00047 | 0.00053 | 0.00057 | 0.00061 |
| PTSD | 0.00059 | 0.00070 | 0.00078 | 0.00084 | 0.00090 |
| Anorexia nervosa | 0.00048 | 0.00057 | 0.00063 | 0.00069 | 0.00073 |

| | | | | | |
|---|---|---|---|---|---|
| **Alcohol daily use** | 0.00150 | 0.00179 | 0.00199 | 0.00216 | 0.00231 |
| N cigarettes per day | 0.00028 | 0.00033 | 0.00037 | 0.00041 | 0.00043 |
| Cannabis use lifetime | 0.00021 | 0.00024 | 0.00027 | 0.00030 | 0.00032 |
| Extraversion | 0.00011 | 0.00013 | 0.00014 | 0.00015 | 0.00016 |
| Neuroticism | 0.00061 | 0.00072 | 0.00080 | 0.00087 | 0.00093 |
| Type 2 diabetes | 0.00061 | 0.00073 | 0.00081 | 0.00088 | 0.00094 |
| **Coronary artery disease** | 0.00143 | 0.00169 | 0.00189 | 0.00205 | 0.00219 |
| **Triglycerides** | 0.00119 | 0.00141 | 0.00157 | 0.00171 | 0.00182 |
| Total LDL cholesterol | 0.00018 | 0.00022 | 0.00024 | 0.00026 | 0.00028 |
| Total HDL cholesterol | 0.00064 | 0.00076 | 0.00085 | 0.00092 | 0.00098 |
| **BMI** | 0.00592 | 0.0070 | 0.00784 | 0.00851 | 0.00909 |
| **Leptin** | 0.00133 | 0.0016 | 0.00177 | 0.00192 | 0.00205 |
| Leptin adjusted for BMI | 0.00011 | 0.00013 | 0.000143 | 0.00016 | 0.00017 |
| **CRP** | 0.00214 | 0.00254 | 0.00284 | 0.00308 | 0.00329 |
| Ischemic stroke | 0.00075 | 0.00089 | 0.00099 | 0.00108 | 0.00115 |

**Supplementary Table 5: A.** PRS results for ↓WS depression (n=10,142) vs. healthy controls (n=8,000) (including bipolar disorder and/or schizophrenia cases, the results were similar when excluding these groups, see Supplementary Figure 4). **B.** Nagelkerke R2 is the reported on the liability scale according to different possible values of ↓WS depression prevalence in the population. Significant results are in bold.

**A**

| Trait | Best p threshold | Beta (SE) | P | OR (95% CI) |
|---|---|---|---|---|
| **Major depressive disorder** | 0.3 | 0.18 (0.01) | 1.95e-62** | 1.20 (1.17-1.23) |
| **Bipolar disorder** | 0.3 | 0.10 (0.01) | 8.66e-20** | 1.11 (1.08-1.13) |
| **Schizophrenia** | 0.5 | 0.13 (0.01) | 2.66e-32** | 1.14 (1.12-1.17) |
| **Anxiety disorders** | 0.5 | 0.06 (0.01) | 1.07e-08** | 1.06 (1.04-1.09) |
| **Alcohol dependence** | 0.5 | 0.06 (0.01) | 2.22e-08** | 1.07 (1.04-1.09) |
| PTSD | 0.4 | 0.03 (0.01) | 1.66e-03* | 1.04 (1.01-1.06) |
| **Anorexia nervosa** | 1 | 0.10 (0.01) | 2.13e-22** | 1.11 (1.09-1.13) |
| Alcohol daily use | 5e-8 | -0.0090 (0.01) | 0.40 | 0.99 (0.97-1.01) |
| N cigarettes per day | 1 | 0.03 (0.01) | 0.01* | 1.03 (1.01-1.05) |
| Cannabis use lifetime | 1 | 0.04 (0.01) | 1.24e-03* | 1.04 (1.01-1.06) |
| Extraversion | 0.01 | -0.02 (0.01) | 0.02* | 0.98 (0.96-1.00) |
| **Neuroticism** | 0.2 | 0.06 (0.01) | 2.50e-07** | 1.06 (1.03-1.08) |
| Type 2 diabetes | 1e-5 | -0.02 (0.01) | 0.05 | 0.98 (0.96-1.00) |
| **Coronary artery disease** | 0.01 | 0.04 (0.01) | 3.65e-05** | 1.05 (1.02-1.07) |
| Triglycerides | 0.5 | 0.04 (0.01) | 9.20e-04* | 1.04 (1.01-1.06) |
| Total LDL cholesterol | 0.001 | 0.02 (0.01) | 0.02* | 1.02 (1.00-1.05) |
| Total HDL cholesterol | 0.1 | -0.02 (0.01) | 0.16 | 0.98 (0.96-1.01) |
| BMI | 1e-5 | -0.04 (0.01) | 1.24e-04* | 0.96 (0.94-0.98) |
| Leptin | 1e-5 | -0.02 (0.01) | 0.11 | 0.98 (0.96-1.00) |
| Leptin adjusted for BMI | 1e-5 | 0.02 (0.01) | 0.16 | 1.02 (0.99-1.04) |
| CRP | 0.5 | 0.02 (0.01) | 0.16 | 1.02 (0.99-1.04) |
| Ischemic stroke | 0.001 | 0.02 (0.01) | 0.03* | 1.02 (1.00-1.05) |

* p < 0.05; ** p < 2.1e-04

**B**

| Trait | K=0.03 | K=0.04 | K=0.05 | K=0.06 | K=0.07 |
|---|---|---|---|---|---|
| **Major depressive disorder** | 0.00584 | 0.00634 | 0.00677 | 0.00715 | 0.00749 |
| **Bipolar disorder** | 0.00173 | 0.00188 | 0.00201 | 0.00212 | 0.00223 |
| **Schizophrenia** | 0.00293 | 0.00318 | 0.00340 | 0.00359 | 0.00377 |
| **Anxiety disorders** | 0.00068 | 0.00074 | 0.00079 | 0.00084 | 0.00087 |
| **Alcohol dependence** | 0.00065 | 0.00071 | 0.00076 | 0.00080 | 0.00084 |
| PTSD | 0.00021 | 0.00022 | 0.00024 | 0.00025 | 0.00026 |
| **Anorexia nervosa** | 0.00195 | 0.00212 | 0.00226 | 0.00239 | 0.00251 |

| | | | | | |
|---|---|---|---|---|---|
| Alcohol daily use | 1.49e-05 | 1.62e-05 | 1.73e-05 | 1.83e-05 | 1.92e-05 |
| N cigarettes per day | 0.00012 | 0.00014 | 0.00014 | 0.00015 | 0.00016 |
| Cannabis use lifetime | 0.00022 | 0.00024 | 0.00025 | 0.00027 | 0.00028 |
| Extraversion | 0.00011 | 0.00012 | 0.00013 | 0.00013 | 0.00014 |
| **Neuroticism** | 0.00056 | 0.00060 | 0.00064 | 0.00068 | 0.00071 |
| Type 2 diabetes | 8.19e-05 | 8.89e-05 | 9.50e-05 | 1.0e-04 | 1.05e-04 |
| **Coronary artery disease** | 0.00036 | 0.00039 | 0.00041 | 0.00044 | 0.00046 |
| Triglycerides | 0.00023 | 0.00025 | 0.00027 | 0.00028 | 0.00029 |
| Total LDL cholesterol | 0.00011 | 0.00012 | 0.00013 | 0.00013 | 0.00014 |
| Total HDL cholesterol | 4.15e-05 | 4.51e-05 | 4.82e-05 | 5.09e-05 | 5.34e-05 |
| BMI | 0.00031 | 0.00033 | 0.00036 | 0.00038 | 0.00040 |
| Leptin | 5.22e-05 | 5.66e-05 | 6.05e-05 | 6.39e-05 | 6.71e-05 |
| Leptin adjusted for BMI | 4.08e-05 | 4.43e-05 | 4.73e-05 | 5.00e-05 | 5.25e-05 |
| CRP | 4.15e-05 | 4.51e-05 | 4.81e-05 | 5.08e-05 | 5.33e-05 |
| Ischemic stroke | 0.00010 | 0.00011 | 0.00012 | 0.00013 | 0.00013 |

**Supplementary Figure 1:** barplots representing the results (p-values and Nagelkerke $R^2$) obtained at the different $P_T$ for the PRS of traits associated with ↑WS depression vs. depression without ↑WS (leptin was very close to the Bonferroni corrected p threshold, the other PRS were statistically significant after Bonferroni correction, see Table 1). Nagelkerke $R^2$ was reported on the observed scale, since these comparisons included cases only. **A.** BMI; **B.** Leptin; **C.** C-reactive protein (CRP); **D.** Daily alcohol use; **E.** Major depressive disorder.

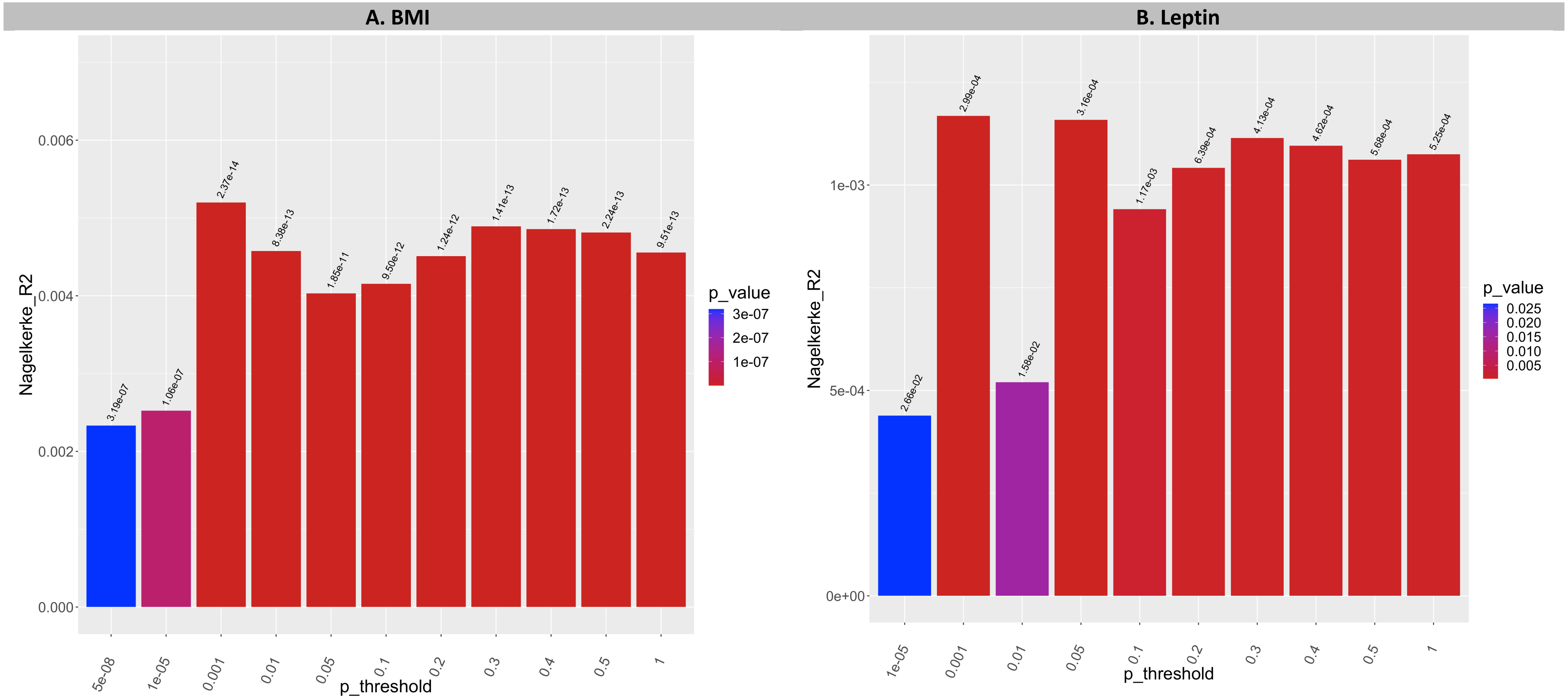

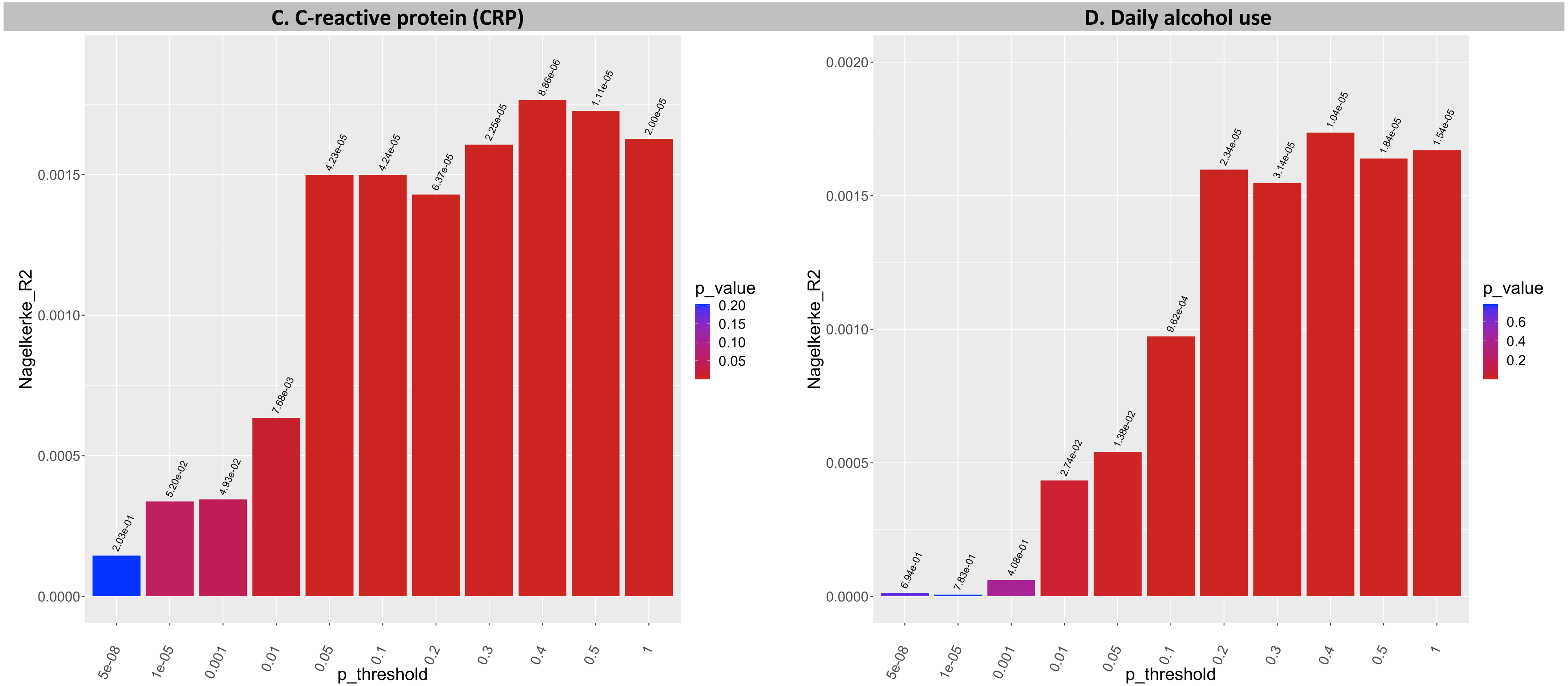
C. C-reactive protein (CRP)
D. Daily alcohol use
Nagelkerke_R2
p_threshold
p_value
0.0000
0.0005
0.0010
0.0015
0.0020
0.20
0.15
0.10
0.05
0.6
0.4
0.2
5e-08
1e-05
0.001
0.01
0.05
0.1
0.2
0.3
0.4
0.5
1
2.03e-01
5.20e-02
4.93e-02
7.68e-03
4.23e-05
4.24e-05
6.37e-05
2.25e-05
8.86e-06
1.11e-05
2.00e-05
6.94e-01
7.83e-01
4.08e-01
2.74e-02
1.38e-02
9.62e-04
2.34e-05
3.14e-05
1.04e-05
1.84e-05
1.54e-05

**E. Major depressive disorder**

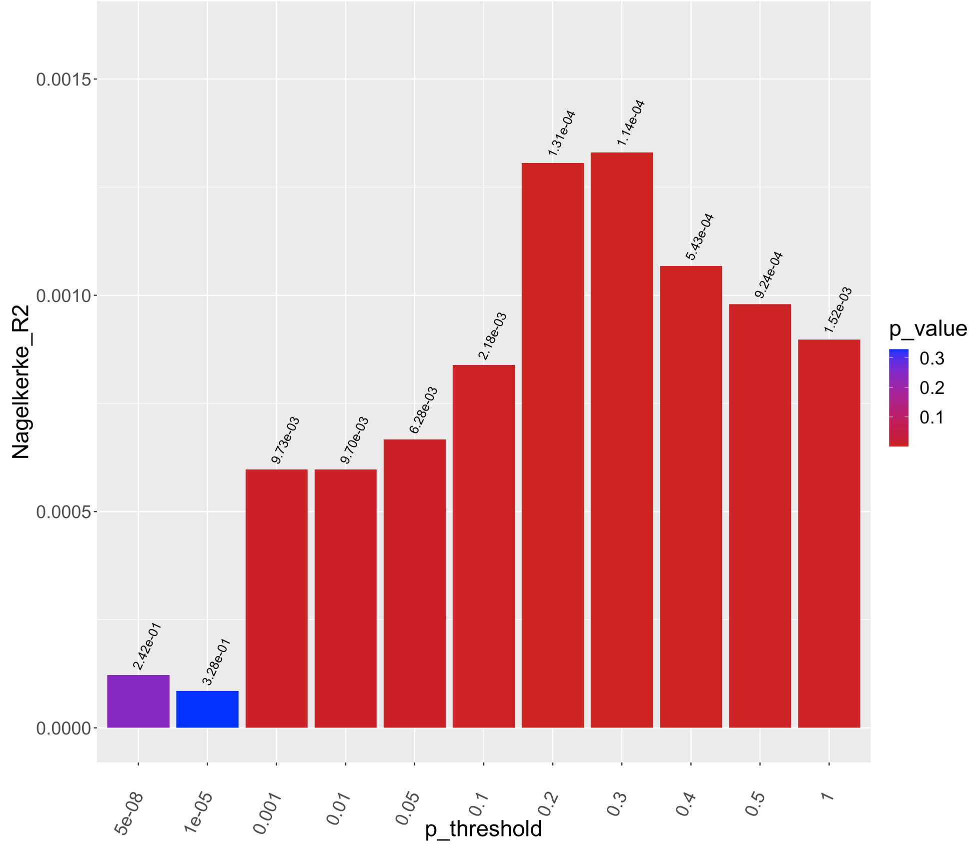

**Supplementary Figure 2:** PRS odds ratio (OR) and 95% confidence intervals of ↑WS depression vs. non-↑WS depression after excluding people with probable bipolar disorder or schizophrenia or missing information for these variables. None of the results was significantly different compared to what observed in the main analysis. Colors indicate different groups of traits (substance related disorders, major psychiatric disorders, personality traits, immune metabolic traits). BP=bipolar disorder; SCZ=schizophrenia; ANX=anxiety disorders; PTSD=post-traumatic stress disorder; AN=anorexia nervosa; ALCDEP=alcohol dependence; ALCUSE=daily alcohol use; N_CIGARETTES=n cigarettes per day; CANN=cannabis use lifetime; EXTR=extraversion; NEU=neuroticism; DM2=type 2 diabetes mellitus; CAD=coronary artery disease; ISCH_STROKE=ischemic stroke; TG=triglycerides; LDL=total LDL cholesterol; HDL=total HDL cholesterol; BMI=body max index; BMI_adjust_leptin=leptin adjusted for BMI; CRP=C-reactive protein.

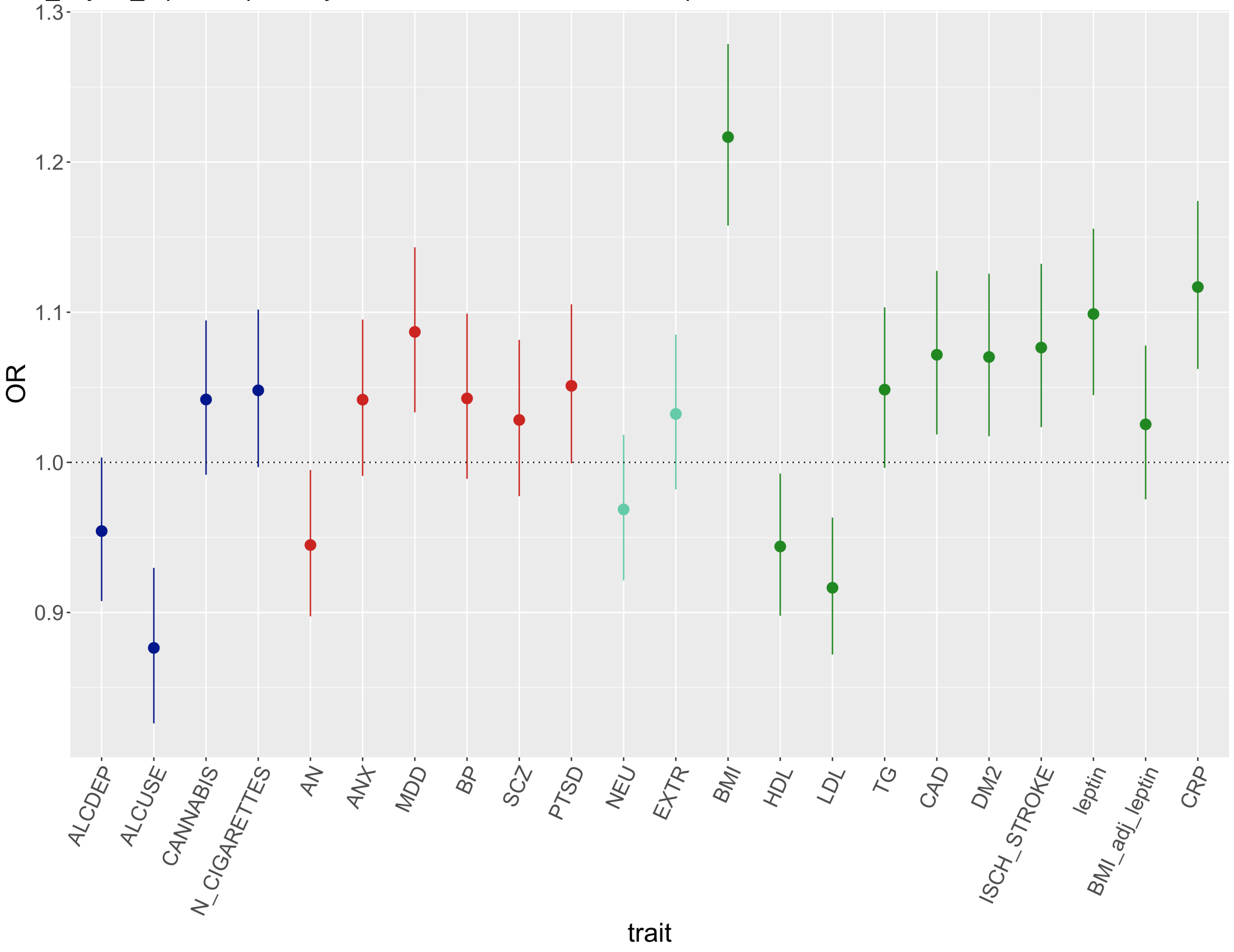

**Supplementary Figure 3:** PRS odds ratio (OR) and 95% confidence intervals of ↑WS depression vs. ↓WS depression after excluding people with probable bipolar disorder or schizophrenia or missing information for these variables. None of the results was significantly different compared to what observed in the main analysis. Colors indicate different groups of traits (substance related disorders, major psychiatric disorders, personality traits, immune metabolic traits). BP=bipolar disorder; SCZ=schizophrenia; ANX=anxiety disorders; PTSD=post-traumatic stress disorder; AN=anorexia nervosa; ALCDEP=alcohol dependence; ALCUSE=daily alcohol use; N_CIGARETTES=n cigarettes per day; CANN=cannabis use lifetime; EXTR=extraversion; NEU=neuroticism; DM2=type 2 diabetes mellitus; CAD=coronary artery disease; ISCH_STROKE=ischemic stroke; TG=triglycerides; LDL=total LDL cholesterol; HDL=total HDL cholesterol; BMI=body max index; BMI_adjust_leptin=leptin adjusted for BMI; CRP=C-reactive protein.

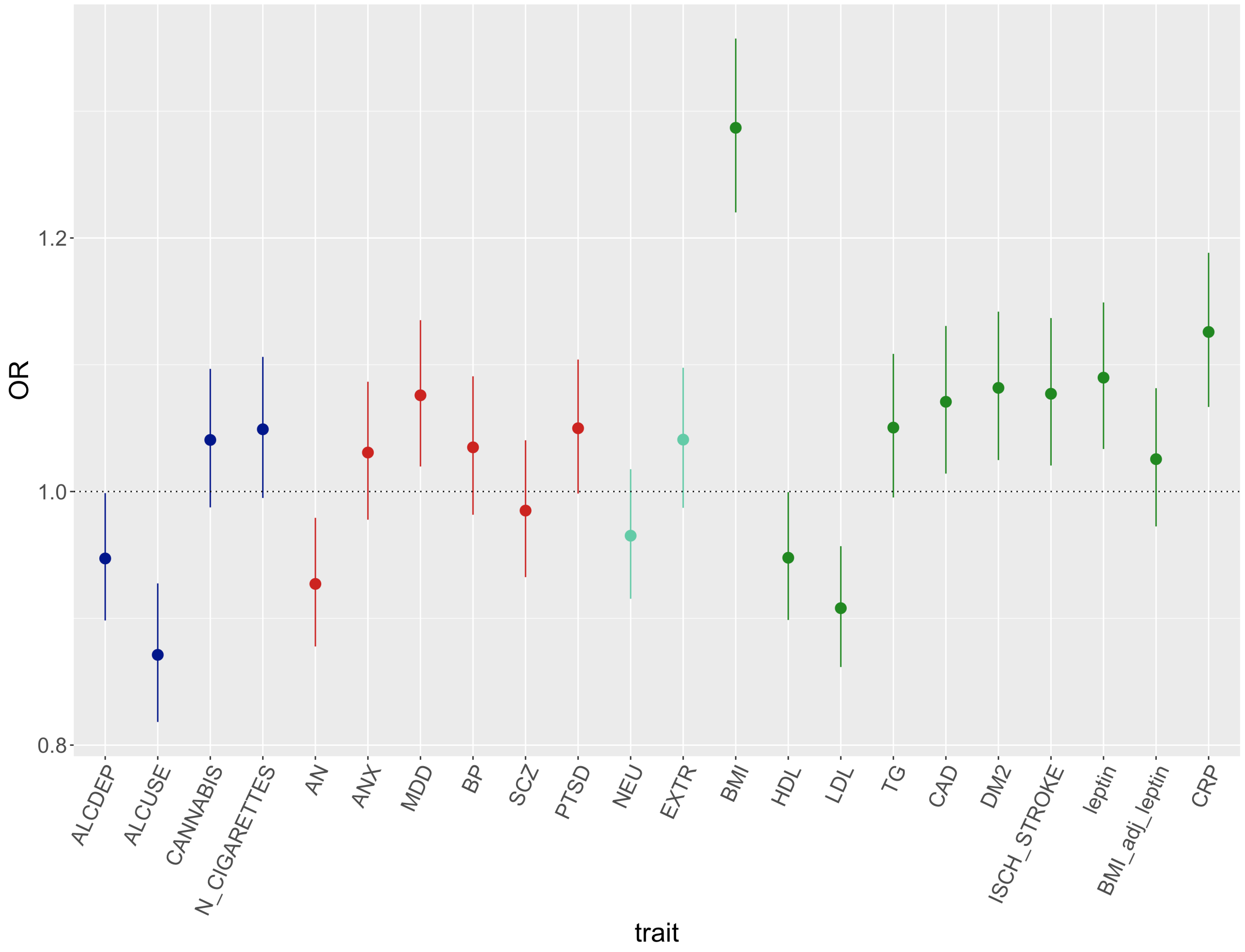

**Supplementary Figure 4:** PRS odds ratio (OR) and 95% confidence intervals of ↑WS depression and ↓WS depression vs. healthy controls after excluding people with probable bipolar disorder or schizophrenia or missing information for these variables. None of the results was significantly different compared to what observed in the main analysis. BP=bipolar disorder; SCZ=schizophrenia; ANX=anxiety disorders; PTSD=post-traumatic stress disorder; AN=anorexia nervosa; ALCDEP=alcohol dependence; ALCUSE=daily alcohol use; N_CIGARETTES=n cigarettes per day; CANN=cannabis use lifetime; EXTR=extraversion; NEU=neuroticism; DM2=type 2 diabetes mellitus; CAD=coronary artery disease; ISCH_STROKE=ischemic stroke; TG=triglycerides; LDL=total LDL cholesterol; HDL=total HDL cholesterol; BMI=body max index; BMI_adjust_leptin=leptin adjusted for BMI; CRP=C-reactive protein.

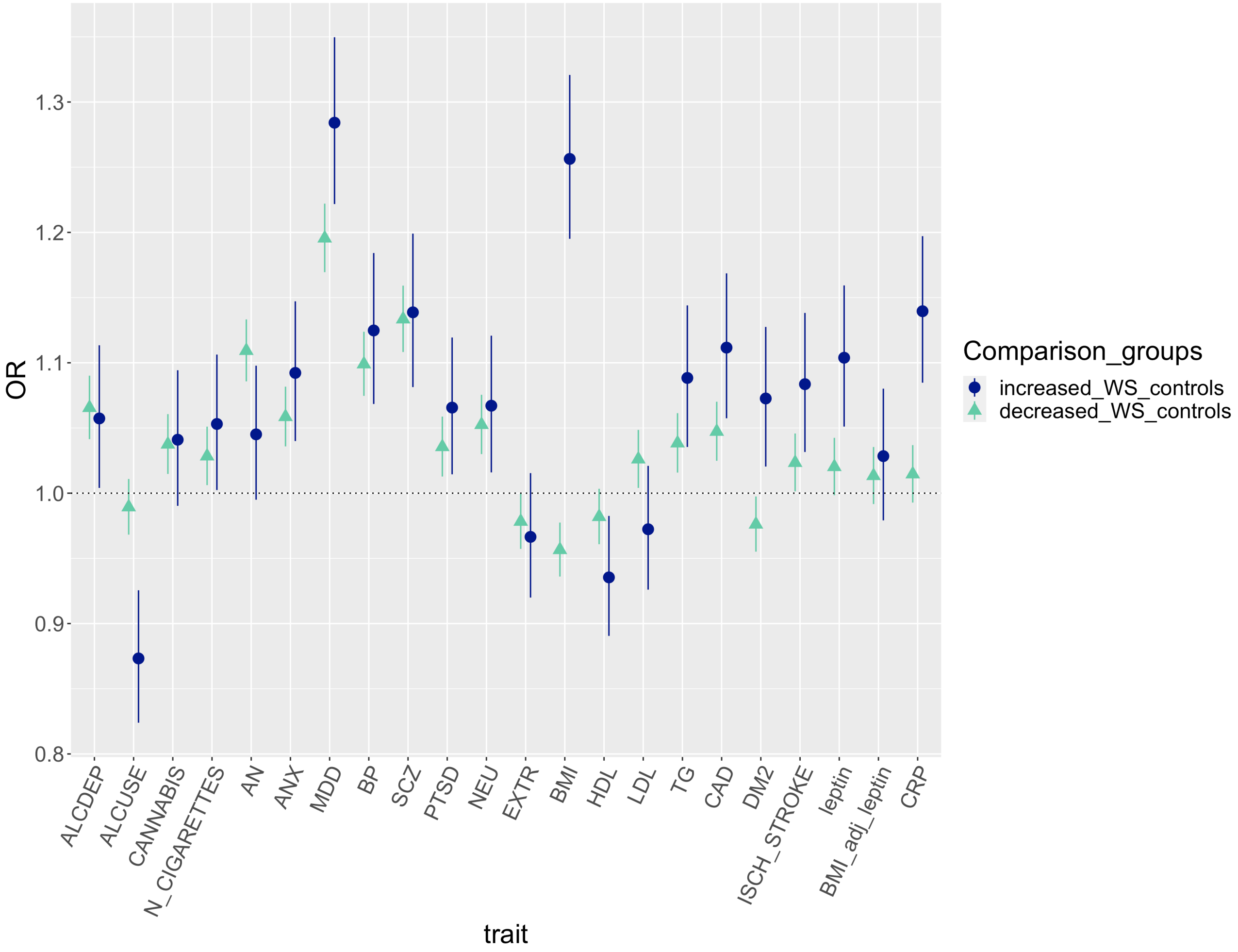

**Supplementary Figure 5:** estimate and 95% confidence intervals calculated by GCTB-Bayes S for S and Pi parameters. S reflects the correlation between minor allele frequency and SNP effect size, negative values are suggestive of negative natural selection; no depression subtype had S significantly different from zero after multiple-testing correction though depression with weight increase showed a S nominally significantly different from zero (p=0.018). Pi is the proportion of SNPs with non-zero effects. WS=weight and sleep.

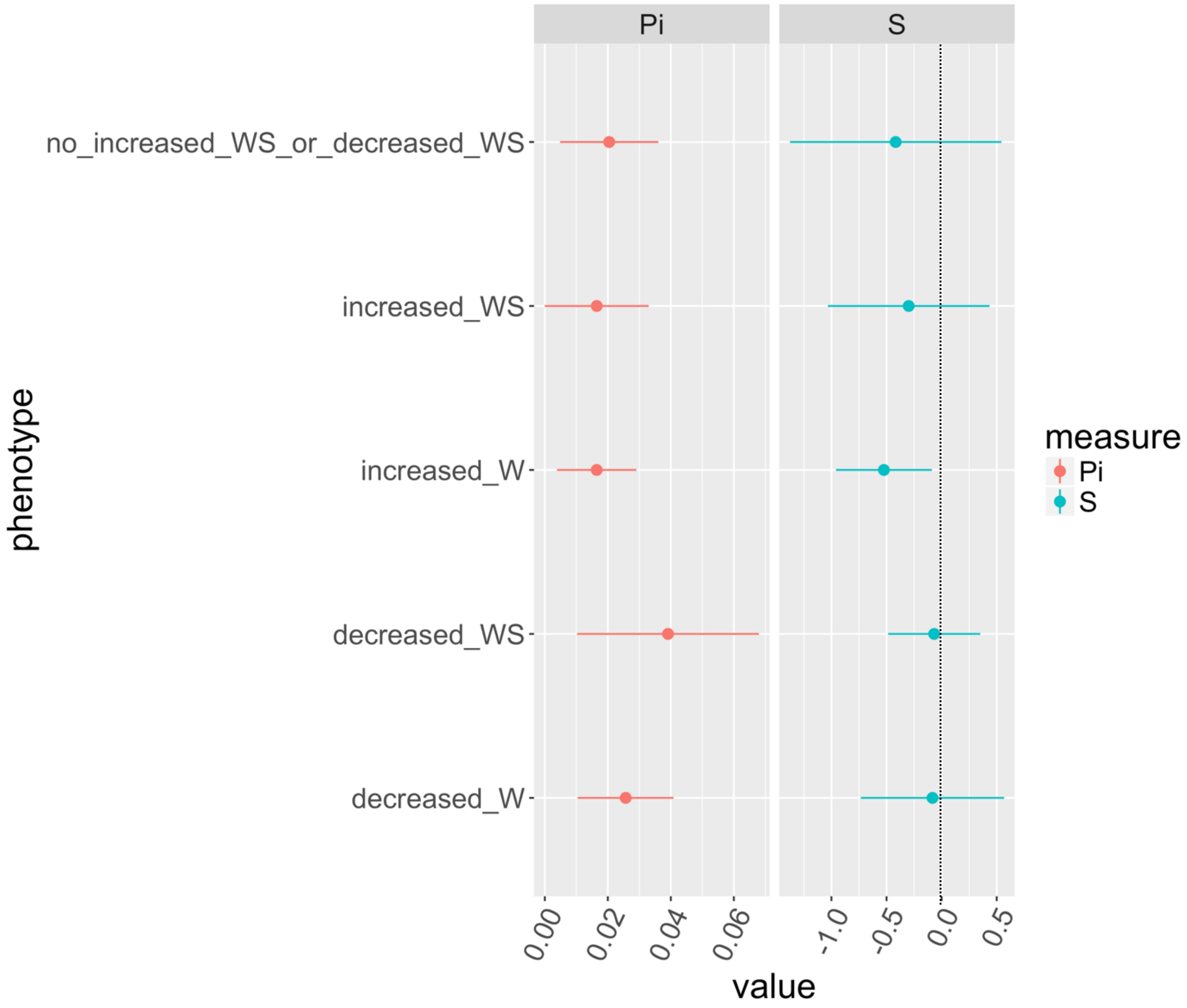

## References


Duncan, L. E., Ratanatharathorn, A., Aiello, A. E., Almli, L. M., Amstadter, A. B., Ashley-Koch, A. E., Baker, D. G., Beckham, J. C., Bierut, L. J., Bisson, J., Bradley, B., Chen, C.-Y., Dalvie, S., Farrer, L. A., Galea, S., Garrett, M. E., Gelernter, J. E., Guffanti, G., Hauser, M. A., … Koenen, K. C. (2018). Largest GWAS of PTSD (N=20 070) yields genetic overlap with schizophrenia and sex differences in heritability. *Molecular Psychiatry*, *23*(3), 666–673. https://doi.org/10.1038/mp.2017.77

Genetics of Personality Consortium, de Moor, M. H. M., van den Berg, S. M., Verweij, K. J. H., Krueger, R. F., Luciano, M., Arias Vasquez, A., Matteson, L. K., Derringer, J., Esko, T., Amin, N., Gordon, S. D., Hansell, N. K., Hart, A. B., Seppälä, I., Huffman, J. E., Konte, B., Lahti, J., Lee, M., … Boomsma, D. I. (2015). Meta-analysis of Genome-wide Association Studies for Neuroticism, and the Polygenic Association With Major Depressive Disorder. *JAMA Psychiatry*, *72*(7), 642–650. https://doi.org/10.1001/jamapsychiatry.2015.0554

Kilpeläinen, T. O., Carli, J. F. M., Skowronski, A. A., Sun, Q., Kriebel, J., Feitosa, M. F., Hedman, Å. K., Drong, A. W., Hayes, J. E., Zhao, J., Pers, T. H., Schick, U., Grarup, N., Kutalik, Z., Trompet, S., Mangino, M., Kristiansson, K., Beekman, M., Lyytikäinen, L.-P., … Loos, R. J. F. (2016). Genome-wide meta-analysis uncovers novel loci influencing circulating leptin levels. *Nature Communications*, *7*, 10494. https://doi.org/10.1038/ncomms10494

Ligthart, S., Vaez, A., Võsa, U., Stathopoulou, M. G., de Vries, P. S., Prins, B. P., Van der Most, P. J., Tanaka, T., Naderi, E., Rose, L. M., Wu, Y., Karlsson, R., Barbalic, M., Lin, H., Pool, R., Zhu, G., Macé, A., Sidore, C., Trompet, S., … Alizadeh, B. Z. (2018). Genome Analyses of >200,000 Individuals Identify 58 Loci for Chronic Inflammation and Highlight Pathways that Link Inflammation and Complex Disorders. *American Journal of Human Genetics*, *103*(5), 691–706. https://doi.org/10.1016/j.ajhg.2018.09.009

Locke, A. E., Kahali, B., Berndt, S. I., Justice, A. E., Pers, T. H., Day, F. R., Powell, C., Vedantam, S., Buchkovich, M. L., Yang, J., Croteau-Chonka, D. C., Esko, T., Fall, T., Ferreira, T., Gustafsson, S., Kutalik, Z., Luan, J., Mägi, R., Randall, J. C., … Speliotes, E. K. (2015). Genetic studies of body mass index yield new insights for obesity biology. *Nature*, *518*(7538), 197–206. https://doi.org/10.1038/nature14177

Malik, R., Chauhan, G., Traylor, M., Sargurupremraj, M., Okada, Y., Mishra, A., Rutten-Jacobs, L., Giese, A.-K., van der Laan, S. W., Gretarsdottir, S., Anderson, C. D., Chong, M., Adams, H. H. H., Ago, T., Almgren, P., Amouyel, P., Ay, H., Bartz, T. M., Benavente, O. R., … Dichgans, M.

(2018). Multiancestry genome-wide association study of 520,000 subjects identifies 32 loci associated with stroke and stroke subtypes. *Nature Genetics*, *50*(4), 524–537. https://doi.org/10.1038/s41588-018-0058-3

Nikpay, M., Goel, A., Won, H.-H., Hall, L. M., Willenborg, C., Kanoni, S., Saleheen, D., Kyriakou, T., Nelson, C. P., Hopewell, J. C., Webb, T. R., Zeng, L., Dehghan, A., Alver, M., Armasu, S. M., Auro, K., Bjonnes, A., Chasman, D. I., Chen, S., … Farrall, M. (2015). A comprehensive 1,000 Genomes-based genome-wide association meta-analysis of coronary artery disease. *Nature Genetics*, *47*(10), 1121–1130. https://doi.org/10.1038/ng.3396

Otowa, T., Hek, K., Lee, M., Byrne, E. M., Mirza, S. S., Nivard, M. G., Bigdeli, T., Aggen, S. H., Adkins, D., Wolen, A., Fanous, A., Keller, M. C., Castelao, E., Kutalik, Z., Van der Auwera, S., Homuth, G., Nauck, M., Teumer, A., Milaneschi, Y., … Hettema, J. M. (2016). Meta-analysis of genome-wide association studies of anxiety disorders. *Molecular Psychiatry*, *21*(10), 1391–1399. https://doi.org/10.1038/mp.2015.197

Ripke, S., Neale, B. M., Corvin, A., Walters, J. T. R., Farh, K.-H., Holmans, P. A., Lee, P., Bulik-Sullivan, B., Collier, D. A., Huang, H., Pers, T. H., Agartz, I., Agerbo, E., Albus, M., Alexander, M., Amin, F., Bacanu, S. A., Begemann, M., Belliveau Jr, R. A., … O'Donovan, M. C. (2014). Biological insights from 108 schizophrenia-associated genetic loci. *Nature*, *511*(7510), 421–427. https://doi.org/10.1038/nature13595

Schumann, G., Liu, C., O'Reilly, P., Gao, H., Song, P., Xu, B., Ruggeri, B., Amin, N., Jia, T., Preis, S., Segura Lepe, M., Akira, S., Barbieri, C., Baumeister, S., Cauchi, S., Clarke, T.-K., Enroth, S., Fischer, K., Hällfors, J., … Elliott, P. (2016). KLB is associated with alcohol drinking, and its gene product β-Klotho is necessary for FGF21 regulation of alcohol preference. *Proceedings of the National Academy of Sciences of the United States of America*, *113*(50), 14372–14377. https://doi.org/10.1073/pnas.1611243113

Scott, R. A., Scott, L. J., Mägi, R., Marullo, L., Gaulton, K. J., Kaakinen, M., Pervjakova, N., Pers, T. H., Johnson, A. D., Eicher, J. D., Jackson, A. U., Ferreira, T., Lee, Y., Ma, C., Steinthorsdottir, V., Thorleifsson, G., Qi, L., Van Zuydam, N. R., Mahajan, A., … DIAbetes Genetics Replication And Meta-analysis (DIAGRAM) Consortium. (2017). An Expanded Genome-Wide Association Study of Type 2 Diabetes in Europeans. *Diabetes*, *66*(11), 2888–2902. https://doi.org/10.2337/db16-1253

Stahl, E. A., Breen, G., Forstner, A. J., McQuillin, A., Ripke, S., Trubetskoy, V., Mattheisen, M., Wang, Y., Coleman, J. R. I., Gaspar, H. A., de Leeuw, C. A., Steinberg, S., Pavlides, J. M. W.,

Trzaskowski, M., Byrne, E. M., Pers, T. H., Holmans, P. A., Richards, A. L., Abbott, L., … Bipolar Disorder Working Group of the Psychiatric Genomics Consortium. (2019). Genome-wide association study identifies 30 loci associated with bipolar disorder. *Nature Genetics*, *51*(5), 793–803. https://doi.org/10.1038/s41588-019-0397-8

Stringer, S., Minică, C. C., Verweij, K. J. H., Mbarek, H., Bernard, M., Derringer, J., van Eijk, K. R., Isen, J. D., Loukola, A., Maciejewski, D. F., Mihailov, E., van der Most, P. J., Sánchez-Mora, C., Roos, L., Sherva, R., Walters, R., Ware, J. J., Abdellaoui, A., Bigdeli, T. B., … Vink, J. M. (2016). Genome-wide association study of lifetime cannabis use based on a large meta-analytic sample of 32 330 subjects from the International Cannabis Consortium. *Translational Psychiatry*, *6*, e769. https://doi.org/10.1038/tp.2016.36

Tobacco and Genetics Consortium. (2010). Genome-wide meta-analyses identify multiple loci associated with smoking behavior. *Nature Genetics*, *42*(5), 441–447. https://doi.org/10.1038/ng.571

van den Berg, S. M., de Moor, M. H. M., Verweij, K. J. H., Krueger, R. F., Luciano, M., Arias Vasquez, A., Matteson, L. K., Derringer, J., Esko, T., Amin, N., Gordon, S. D., Hansell, N. K., Hart, A. B., Seppälä, I., Huffman, J. E., Konte, B., Lahti, J., Lee, M., Miller, M., … Boomsma, D. I. (2016). Meta-analysis of Genome-Wide Association Studies for Extraversion: Findings from the Genetics of Personality Consortium. *Behavior Genetics*, *46*(2), 170–182. https://doi.org/10.1007/s10519-015-9735-5

Walters, R. K., Polimanti, R., Johnson, E. C., McClintick, J. N., Adams, M. J., Adkins, A. E., Aliev, F., Bacanu, S.-A., Batzler, A., Bertelsen, S., Biernacka, J. M., Bigdeli, T. B., Chen, L.-S., Clarke, T.-K., Chou, Y.-L., Degenhardt, F., Docherty, A. R., Edwards, A. C., Fontanillas, P., … Agrawal, A. (2018). Transancestral GWAS of alcohol dependence reveals common genetic underpinnings with psychiatric disorders. *Nature Neuroscience*, *21*(12), 1656–1669. https://doi.org/10.1038/s41593-018-0275-1

Watson, H. J., Yilmaz, Z., Thornton, L. M., Hübel, C., Coleman, J. R. I., Gaspar, H. A., Bryois, J., Hinney, A., Leppä, V. M., Mattheisen, M., Medland, S. E., Ripke, S., Yao, S., Giusti-Rodríguez, P., Anorexia Nervosa Genetics Initiative, Hanscombe, K. B., Purves, K. L., Eating Disorders Working Group of the Psychiatric Genomics Consortium, Adan, R. A. H., … Bulik, C. M. (2019). Genome-wide association study identifies eight risk loci and implicates metabo-psychiatric origins for anorexia nervosa. *Nature Genetics*, *51*(8), 1207–1214. https://doi.org/10.1038/s41588-019-0439-2

Willer, C. J., Schmidt, E. M., Sengupta, S., Peloso, G. M., Gustafsson, S., Kanoni, S., Ganna, A., Chen, J., Buchkovich, M. L., Mora, S., Beckmann, J. S., Bragg-Gresham, J. L., Chang, H.-Y., Demirkan, A., Den Hertog, H. M., Do, R., Donnelly, L. A., Ehret, G. B., Esko, T., … Global Lipids Genetics Consortium. (2013). Discovery and refinement of loci associated with lipid levels. *Nature Genetics*, *45*(11), 1274–1283. https://doi.org/10.1038/ng.2797

Wray, N. R., Ripke, S., Mattheisen, M., Trzaskowski, M., Byrne, E. M., Abdellaoui, A., Adams, M. J., Agerbo, E., Air, T. M., Andlauer, T. M. F., Bacanu, S.-A., Bækvad-Hansen, M., Beekman, A. F. T., Bigdeli, T. B., Binder, E. B., Blackwood, D. R. H., Bryois, J., Buttenschøn, H. N., Bybjerg-Grauholm, J., … Major Depressive Disorder Working Group of the Psychiatric Genomics Consortium. (2018). Genome-wide association analyses identify 44 risk variants and refine the genetic architecture of major depression. *Nature Genetics*, *50*(5), 668–681. https://doi.org/10.1038/s41588-018-0090-3
